# Accuracy of multimodal vaginal ultrasound in the detection and assessment of scar healing after caesarean section: a correlational meta-analysis

**DOI:** 10.1080/07853890.2025.2523558

**Published:** 2025-06-30

**Authors:** Li Qiao, Jingjing Lu, Bihong Zhang, Aiqun Liu, Xiaohua Wang, Mei Wang, Jian Li, Jiabao Meng

**Affiliations:** a Department of ultrasound, Zhongshan Boai Hospital, Zhongshan, China; b Department maternity, Shenzhen Luohu District People’s Hospital, Shenzhen, China; c Department of Science and Education, Zhongshan Boai Hospital, Zhongshan, China; d Department Organization and Personnel, Zhongshan Boai Hospital, Zhongshan, China

**Keywords:** Multimodal vaginal ultrasound, caesarean section, scar healing, diagnosis, META

## Abstract

**Objective:**

To evaluate the accuracy of multimodal vaginal ultrasound in assessing post-caesarean scar healing.

**Methods:**

A systematic review and meta-analysis were conducted on Chinese and English studies using multimodal vaginal ultrasound to diagnose poor scar healing post-CS. Two researchers screened literature based on inclusion/exclusion criteria. Quality assessment and meta-analysis (RevMan 5.4, Stata14.0, Meta-DiSc1.4) were performed for various diagnostic indicators.

**Results:**

Twenty-five high-quality studies identified key ultrasound markers: hypoechoic/anechoic scars (sensitivity 92%, specificity 91%), thinning/discontinuity of the myotomy layer (sensitivity 95%, specificity 90%), blurred incision contour (sensitivity 99%, specificity 91%), absent blood flow (sensitivity 92%, specificity 91%), irregular lesion morphology (sensitivity 95%, specificity 90%), uneven myometrial echogenicity (sensitivity 94%, specificity 91%), lower uterine segment thickness ≤3.73 mm (sensitivity 90%, specificity 88%), and myometrial lining ≤1.5 mm (sensitivity 90%, specificity 92%).

**Conclusion:**

Multimodal vaginal ultrasound is highly accurate in detecting poor CS scar healing, aiding early intervention to prevent complications.

## Introduction

The incidence of cesarean section surgeries has been steadily increasing over the years. Consequently, the detection and evaluation of uterine scar healing status following cesarean delivery have garnered significant attention from the medical community. [1]. Poor scar healing, often referred to in medical terminology as ‘post-cesarean section uterine scar defects’ [2] or ‘incomplete healing of uterine incision’ [3], not only affects the reproductive health and fertility of the mother, but also increases the risks of second pregnancies such as uterine rupture [4], uterine bleeding [5], uterine bleeding [6], uterine bleeding [7], uterine bleeding [8], uterine bleeding [9], and uterine bleeding [11], placenta praevia and other serious complications [5]. Therefore, the exploration of an accurate and non-invasive testing technique holds significant medical value for the precise assessment of scar healing following cesarean section. This is essential not only for ensuring maternal physical and mental well-being but also for enhancing the safety of subsequent pregnancies.

To accurately evaluate the status of scar healing, multimodal transvaginal ultrasound (MTVUS), an emerging non-invasive detection technique, has gained widespread application in the field of obstetrics and gynecology [6]. MTVUS has been widely used in the field of obstetrics and gynaecology by integrating two-dimensional grey-scale ultrasound [7], three-dimensional ultrasonography [8], colour Doppler blood flow imaging [9] and energy Doppler and other advanced imaging techniques [10], this advanced imaging technique is capable of presenting a comprehensive and multi-faceted visualization of the anatomical structure and physiological characteristics of the uterus, particularly focusing on the scar region. Globally, the application of Multi-transducer Three-dimensional Vaginal Ultrasound (MTVUS) in evaluating the scarring aftermath of caesarean section has already undergone partial exploration. In comparison to traditional 2D ultrasound, 3D ultrasound technology offers a distinct advantage in precisely assessing scar defects and diverticula, thereby significantly enhancing the diagnostic accuracy for medical professionals [11]. For scar uterus, the high incidence of chronic endometritis is accompanied by excessive proliferation of fibroblasts in the lower uterine segment and significant increase of vascular *in situ* hyperplasia, while inflammatory factors IL-1α, IL-1β, IL-6, IL-8, TNF-α, and SDF-1α in scar diverticulum (CSD) are elevated [12]. The Doppler technique is valuable in assessing blood flow status in the scarred area, especially in predicting the risk of uterine rupture. A study using MTVUS to predict spontaneous preterm birth in low-risk women at 20 to 24 weeks gestation showed that the ultrasound-related predictors were well discriminated (C-index = 0.898 and 0.839) and calibrated (*p* = 0.258 and 0.115) [13]. Studies have shown that MTVUS can be used for ovarian cancer screening, using longitudinal CA125 algorithm, repeated CA125 test and transvaginal scan (TVS) as a second-line test, but it has a certain false positive rate [14]. However, although MTVUS has shown great potential in the assessment of scarring after caesarean section, results of studies on its accuracy and reliability still vary [15]. Some studies have noted that the diagnostic efficacy of MTVUS may be affected by factors such as patient size, intestinal gas interference, operator experience and skill level [16]. Obese patients have increased adipose tissue and decreased pneumoperitoneum and gastrointestinal filling, which may lead to errors in ultrasound results. Lean patients with reduced muscle mass and prominent bones may also affect the results. The technology of ultrasound technician is not skilled enough, it is difficult to grasp the manifestation, and it is easy to misdiagnose or miss the diagnosis. An analysis of years of guided ultrasound screening results from the UK Ovarian Cancer Screening Collaborative Trial found that 522 (1.0%) of 50,625 patients diagnosed with fallopian tube or ovarian cancer in the multimodal screening (MMS) group of 202,562 postmenopausal women aged 50–74 years enrolled in the analysis. ultrasound screening (USS) included 517 (1.0%) of 50,623 people and 1,016 (1.0%) of 10,314 people in the unscreened group. There was no significant difference between the detection rates of MMS and USS [17]. In addition, methodological differences, sample sizes and quality control standards between studies may also lead to inconsistent results [18].

In view of this, the aim of this study was to assess the diagnostic efficacy of MTVUS in the detection of scar healing after caesarean section by integrating research data from the existing literature through systematic evaluation and META analysis. Through searching medical databases and literature screening, this study obtained literature on scar healing after cesarean section with complete MTVUS data, and extracted diagnostic data, such as the echo pattern of the scar region, blood flow signal distribution, integrity of the muscular layer and its thickness, this study will provide more accurate clinical diagnostic criteria and guidance, thus playing an important role in safeguarding maternal health and improving the safety of second pregnancies.

## 2. Information and methods

### 2.1. Literature search

The literature search method of this paper mainly follows the principles of systematic and comprehensive, and the keywords of ‘multimodal vaginal ultrasound’, ‘cesarean section’ and ‘scar healing’ were identified, and the keywords were searched in the major databases with single or combined keywords. The keywords were identified as ‘multimodal vaginal ultrasound’, ‘cesarean section’ and ‘scar healing’, and the keywords were searched in all major databases with a single or a combination of keywords. The search strategy for the database was ((multimodal transvaginal ultrasound)AND((Cesarean Section) OR (Cesarean) OR (Caesarean section) OR (Caesarean) AND ((scar healing) OR (scar)). The search platform mainly includes PubMed, Embase, Cochrane Library and other authoritative databases in the medical field. During the search process, the time period was set from April 2020 to the present, and Boolean operators were used to connect different keywords to expand the search scope. Since the identification and removal of scar after cesarean section has always been a concern of mothers and obstetricians, the corresponding surgical methods, suture techniques and MTVUS research were also constantly developing. Therefore, the older literature cannot directly provide valuable guidance for current clinical diagnosis and treatment. So we chose the most recent literature after 2020 for our meta-analysis.

### 2.2. Literature inclusion and exclusion criteria

Inclusion criteria: (a) the type of included studies should be original studies, including randomised controlled trials (RCTs), non-randomised controlled trials, cohort studies, case-control studies, or cross-sectional studies; (b) the main study was on postoperative uterine scar healing; (c) the use of multimodal vaginal ultrasound as the main detection or assessment tool; (d) pathological examination or surgery as the gold standard for uterine scarring; (e) The literature should contain clear indicators for ultrasound assessment of scar healing, such as scar size, morphology, and echogenicity; (f) The literature should provide sufficient information on the data for meta-analysis or extraction of key information. (g) Considering the feasibility and accuracy of the study, only English and Chinese literature were included.

Exclusion criteria: (a) exclude duplicate publications or literature with highly similar content; (b) too small sample size; (c) systematic assessment, case reports, conference proceedings, non-clinical studies, Meta-analysis; (d) animal experiments.

### 2.3. Literature screening and data extraction

According to the search protocol, duplicate literature was removed, titles and abstracts of preliminary search literature were read, and literature that clearly did not meet the requirements were excluded; then the remaining literature was read in full text to further determine whether it met the inclusion criteria. Conduct quality assessment of the included literature, checking its research methodology, data collection and analysis process, etc. to ensure its reliability and validity, and finalise the list of literature to be included in the study based on the results of quality assessment and screening. Prepare the data extraction form and specify the information points to be extracted, including the type of ultrasound, examination time, diagnostic effect and other key data. Read the included literature one by one and extract the relevant information according to the data extraction form or template, maintain the accuracy and completeness of the data during the extraction process, and double-check or contact the original authors of the original text for confirmation of the key data or uncertain data. The selection process was carried out independently by two researchers, and all the researchers in this study made the final judgment on the selected literature and obtained the included literature.

### 2.4. Statistical analysis

This study used Stata14.0 and Meta-DiSc1.4 software to statistically analyse the data. The Cochrane Q test was used to assess whether there was heterogeneity between studies caused by non-threshold effects, and if there was significant heterogeneity, the random effects model was selected to combine effect sizes, and the existence of threshold effects was tested by calculating the Spearman correlation coefficient. Q statistics and I^2^ statistics were used to test the heterogeneity of the included literature. The sensitivity analysis of the heterogeneity of the included literatures was carried out using the one-by-one exclusion method in the random effects model, and the remaining literatures were evaluated by one-by-one exclusion. This approach may identify key studies that have a significant impact on the results. The combined effect sizes, including sensitivity and specificity, were extracted from the four-cell table data and forest plots were drawn, and in order to explore the source of heterogeneity, meta-regression analyses were performed, and subgroup analyses were conducted based on the characteristics of the literature. The stability of the combined effect sizes was assessed by sensitivity analysis, and publication bias was assessed using Deek’s funnel plot. Fagan plots were drawn to analyse the clinical value of multimodal vaginal ultrasound in the assessment of scar healing after caesarean section.

## 3. Results

### 3.1. Results of literature inclusion

**Figure 1.**
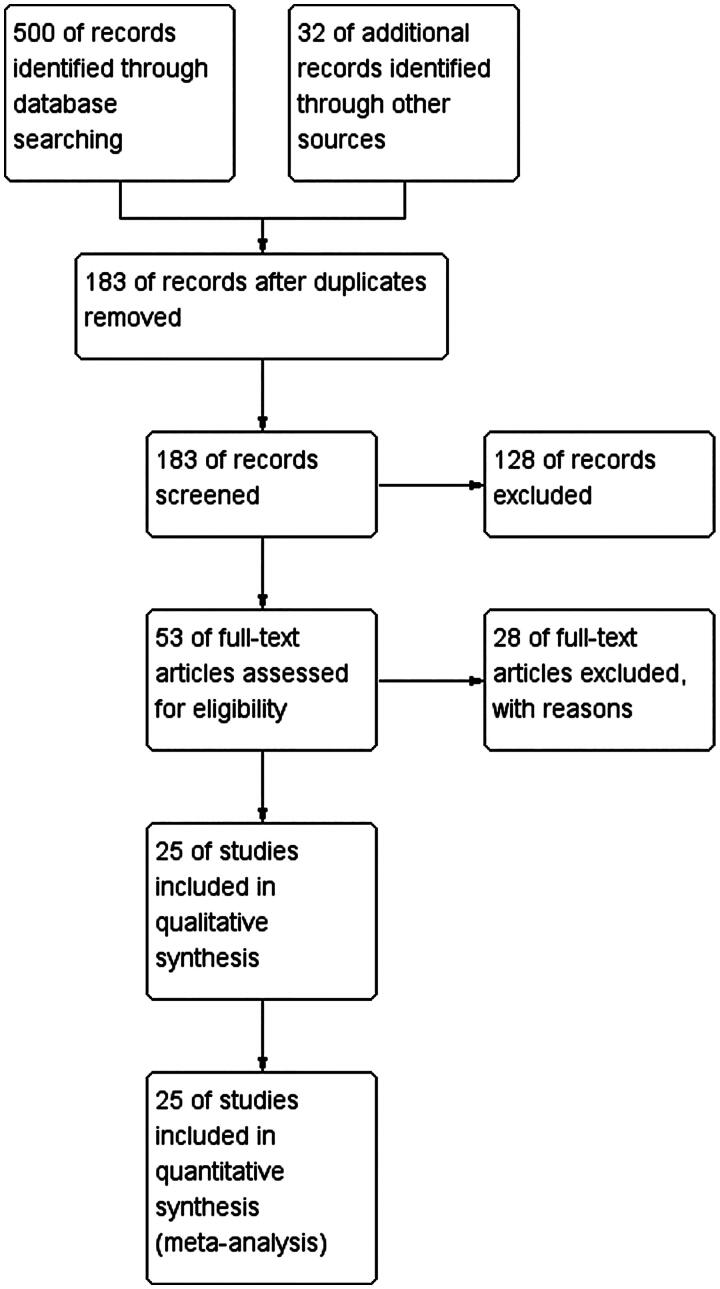
Literature inclusion process.

In this study, 532 pieces of literature were successfully collected from various academic data platforms through database searches. After removing duplicates, 183 pieces of literature remained for further screening. After careful reading of titles and abstracts, 25 reviews, 100 studies not related to the study topic, and 5 animal studies were excluded. After a full-text fine screening of the remaining 53 papers, 10 lower-quality studies, 8 non-randomised controlled studies, and 10 studies that did not meet the outcome metrics were excluded. Ultimately, 25 high-quality studies that were relevant to the study topic were included in the analyses that As shown in Figure 1.

#### 3.1.1. General characteristics of the literature

A total of 25 studies were included, and the study design included prospective and retrospective, with surgical or pathological diagnosis as the gold standard to confirm the diagnosis of uterine scarring. A total of 5061 patients were included, of which 2481 had poorly healed uterine scarring, as shown in Table 1

**Table 1.** General characteristics of the included literature.

Literature	Research Design	TP*	FP*	FN*	TN*	Ultrasound type	Gold standard	Diagnostic indicators
Alalaf 2022 [19]	Prospective	56	9	3	72	Doppler transvaginal ultrasound	operation	1、2、3、4、5、6、7
Alessandri 2023 [20]	Retrospective	80	13	8	90	Transvaginal ultrasound	pathology	1、3、4、5、7
Allegrini 2022 [21]	Prospective	108	22	20	161	Transvaginal ultrasound	pathology	1、3、4、6、7
Chen 2021 [22]	Prospective	55	2	1	70	Transvaginal ultrasound	pathology	1、3、4、7
Chong 2022 [23]	Prospective	236	14	10	232	Doppler transvaginal ultrasound	pathology	1、2、4、5、8
Cohen 2023 [24]	Prospective	81	21	15	98	Transvaginal ultrasound	pathology	1、3、4、7
Di 2023 [25]	Retrospective	75	15	10	68	Transvaginal ultrasound	operation	1、2、4、5
Edwards 2022 [26]	Retrospective	54	4	2	60	Transvaginal ultrasound	operation	1、2、3、4
Glenn 2021 [27]	Retrospective	89	9	13	88	Transvaginal ultrasound	operation	1、3、4、6、8
Jauniaux 2023 [28]	Retrospective	31	3	1	52	Transvaginal ultrasound	operation	1、3、4、5
Jordans 2022 [29]	Retrospective	228	24	44	527	Doppler transvaginal ultrasound	operation	1、3、4、7
Kawakami 2023 [30]	Prospective	78	6	5	34	Transvaginal ultrasound	operation	1、2、3、4、8
Klein 2023 [31]	Prospective	129	23	11	107	Transvaginal ultrasound	pathology	1、3、4、6
Marchant 2023 [32]	Retrospective	76	5	7	79	Transvaginal ultrasound	pathology	1、3、4、5
Mohr-Sasson 2023 [33]	Prospective	75	9	9	83	Transvaginal ultrasound	pathology	1、3、4、6
Morton 2023 [34]	Prospective	94	8	5	65	Transvaginal ultrasound	pathology	1、2、3、4、8
Paping 2023 [35]	Retrospective	63	6	3	43	Transvaginal ultrasound	pathology	1、3、4、5
Qi 2022 [36]	Retrospective	78	7	2	67	Transvaginal ultrasound	operation	1、3、4、5
Risager 2022 [37]	Prospective	74	9	3	48	Transvaginal ultrasound	operation	1、2、3、4、8
Sasaoka 2023 [38]	Prospective	60	4	1	44	Transvaginal ultrasound	operation	1、2、4、7
Savukyne 2022 [39]	Prospective	82	3	1	63	Transvaginal ultrasound	operation	1、2、3、4、8
Shi 2022 [40]	Prospective	95	8	3	65	Transvaginal ultrasound	operation	1、3、4、7
Xiang 2022 [41]	Retrospective	88	10	5	64	Transabdominal ultrasound	pathology	1、2、3、4、8
Zhou 2021 [42]	Prospective	58	3	1	47	Doppler transvaginal ultrasound	pathology	1、2、3、4、5、7
Zhu 2021 [43]	Retrospective	94	7	4	66	Transvaginal ultrasound	pathology	1、3、4、6、8

Table 1: Among the diagnostic indicators, 1 hypoechoic or anechoic, 2 thinning of the myometrium at the incision, discontinuity of the myometrium, fracture defect, 3 blurred outline of the incision, 4 absence of peripheral blood flow signals, 5 irregular morphology of the lesion, 6 partial enhancement of the myometrium echogenicity of the lower segment of the uterus, with uneven thickness, 7 thickness of the lower segment of the uterus ≤3.73 mm, and 8 thickness of the myometrium endometrium ≤1.5 mm. *: TP, true positive, the predicted diagnosis was positive, and the actual diagnosis was positive. FP, false positive, the predicted diagnosis was positive, but the actual diagnosis was negative. FN, false negative, the predicted diagnosis was negative, but the actual diagnosis was positive. TN, true negative, the predicted diagnosis was negative, but the actual diagnosis was negative.

#### 3.1.2. Evaluation of literature quality

**Figure 2.**
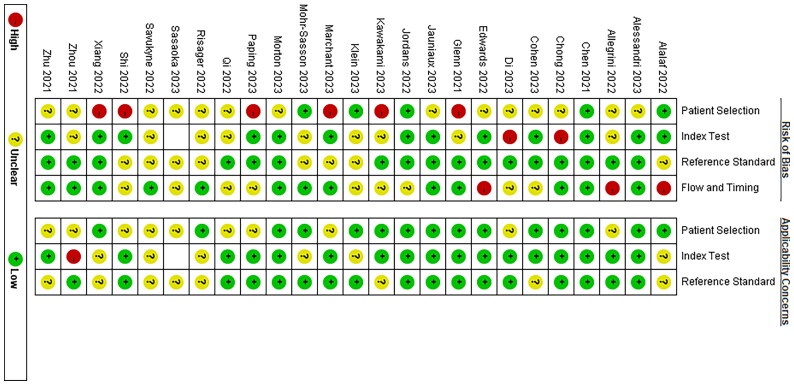
Literature quality assessment.

As shown in Figure 2, there were few included literature that were high risk, and the overall quality of literature included in this study was high.

### 3.2. Meta-analysis results

#### 3.2.1. Heterogeneity test and threshold effect

The Spearman correlation coefficient was calculated with Meta-DiSc1.4 software as 0.244, *p* = 0.261 > 0.05, indicating that there was no threshold effect and effect sizes could be combined.

**Figure 3.**
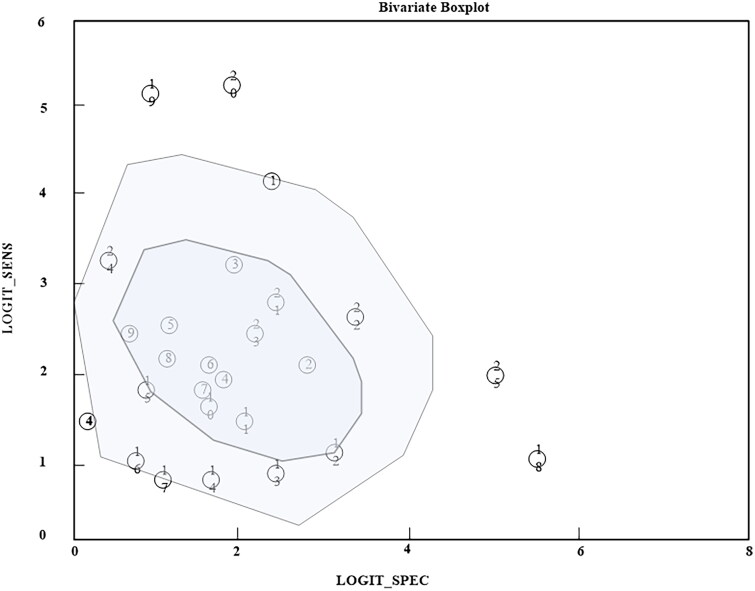
Bivariate box diagram.

Using stata14.0 software to test for heterogeneity, the guard value of sensitivity between the original studies was 83.00% (*p* ≤ 0.01), and the guard value of specificity was 92.33% (*p* < 0.01), both of which showed significant heterogeneity, and should be combined using a random-effects model for the effect sizes. The bivariate box plot shown in Figure 3 shows that some of the scatters are located outside of the centre region and the confidence line, further suggesting that there is heterogeneity among the studies.

The Spearman correlation coefficient = 0.265 was analysed and there was no threshold effect. The results of the heterogeneity test showed that the sensitivity between the original studies was 83.00% (*p* ≤ 0.01) and the specificity was 92.33% (*p* < 0.01), both of which showed significant heterogeneity; therefore, the random effects model was used to combine the effect sizes.

#### 3.2.2. META analysis of the accuracy of multimodal vaginal ultrasound performance in diagnosing scar healing after caesarean section

**Figure 4.**
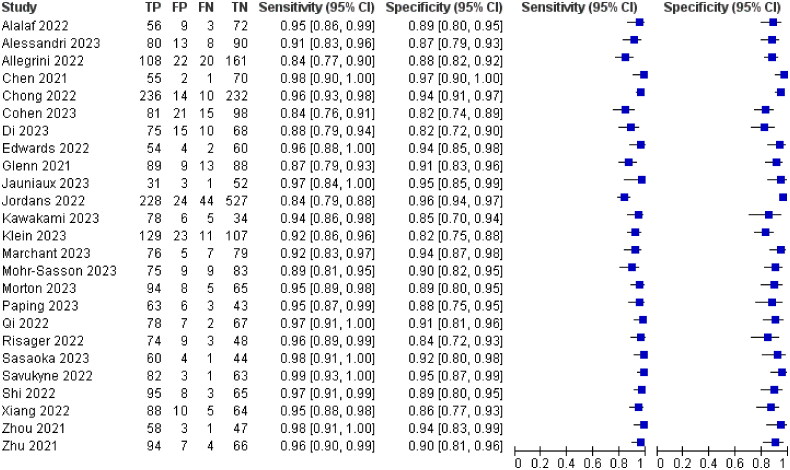
Hypoechoic or non-echoic diagnostic Forest map.

All 25 included papers mentioned hypoechoic or anechoic pregnancy scar as a diagnostic criterion for poor scar healing, with a combined analysis of 92% sensitivity, 95% CI (0.91–0.93) and 91% specificity, 95% CI (0.90–0.92), The results are shown in Figure 4.

**Figure 5.**
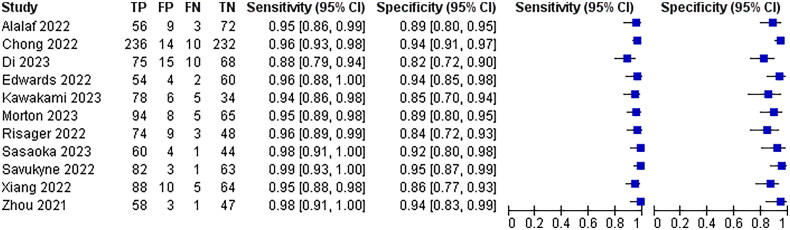
Diagnostic forest plot of muscle thinning at incisions.

Eleven of the 25 papers included mentioned thinning of the myotome at the incision, myotome discontinuity, and fracture defects as diagnostic criteria for poor scar healing, with a combined analysis of 95% sensitivity, 95% CI (0.94–0.97) and 90% specificity, 95% CI (0.88–0.92)The results are shown in Figure 5.

**Figure 6.**
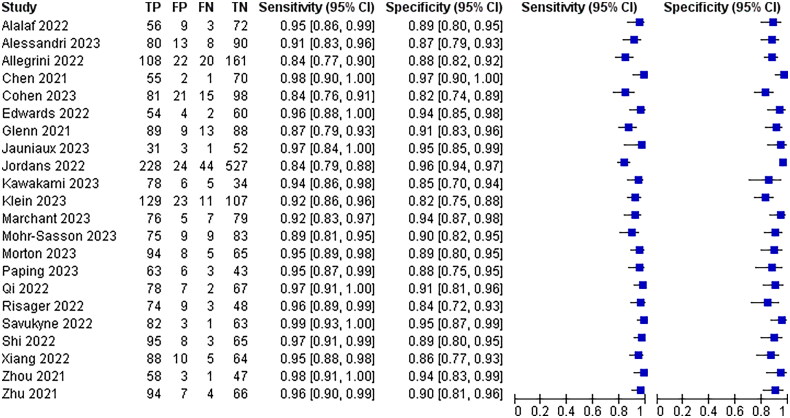
Forest Map for fuzzy diagnosis of incision contour.

Twenty-two of the 25 papers included mentioned blurred incision contour as a diagnostic criterion for poor scar healing, with a combined analysis of 99% sensitivity, 95% CI (0.98–0.99) and 91% specificity, 95% CI (0.89–0.92)The results are shown in Figure 6.

**Figure 7.**
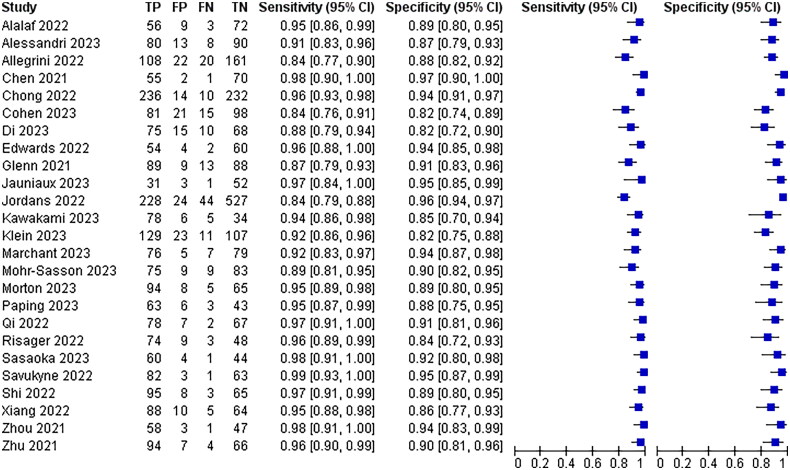
Diagnostic forest plot of peripheral no blood flow signals.

All 25 papers included mentioned the absence of blood flow signal around the scar as a diagnostic criterion for poor scar healing, and the combined analysis had a sensitivity of 95% with a 95% CI (0.94–0.97) and a specificity of 90% with a 95% CI (0.88–0.92)The results are shown in Figure 7.

**Figure 8.**
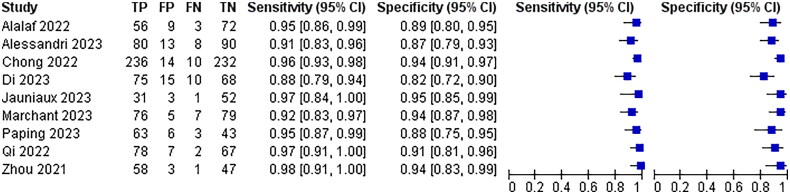
Diagnostic forest plot of irregular lesion morphology.

Irregular lesion morphology was mentioned as a diagnostic criterion for poor scar healing in 9 of the 25 papers included, and the combined analysis had a sensitivity of 94%, 95% CI (0.93–0.96) and a specificity of 91%, 95% CI (0.89–0.93)The results are shown in Figure 8.

**Figure 9.**
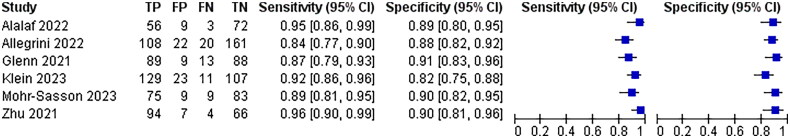
Diagnostic forest plot of partial enhancement of lower segmental muscle echoes.

Six of the 25 papers included mentioned partial enhancement of myometrial echoes in the lower uterine segment with uneven thickness as a diagnostic criterion for poor scar healing, with a combined analysis of 90% sensitivity, 95% CI (0.88–0.92) and 88% specificity, 95% CI (0.85–0.90)The results are shown in Figure 9.

**Figure 10.**
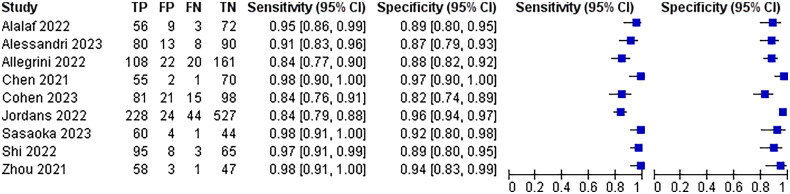
Diagnostic forestart of lower uterine segment thickness ≤3.73 mm.

Nine of the 25 included papers mentioned a lower uterine segment thickness of ≤3.73 mm as a diagnostic criterion for poor scar healing, and the combined analysis had a sensitivity of 90% with a 95% CI (0.87–0.91) and a specificity of 92% with a 95% CI (0.90–0.93)The results are shown in Figure 10.

**Figure 11.**
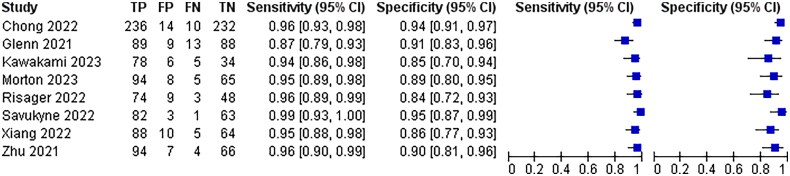
Diagnostic forest plot of myometrial endometrial thickness ≤1.5 mmThe different ultrasound signs were summarised and combined SROC curves are shown in Figure 12.

Eight of the 25 papers included mentioned myometrial endometrial thickness ≤1.5 mm as a diagnostic criterion for poor scar healing, and the combined analysis had a sensitivity of 95% with a 95% CI (0.93–0.96) and a specificity of 91% with a 95% CI (0.89–0.93)The results are shown in Figure 11.

**Figure 12.**
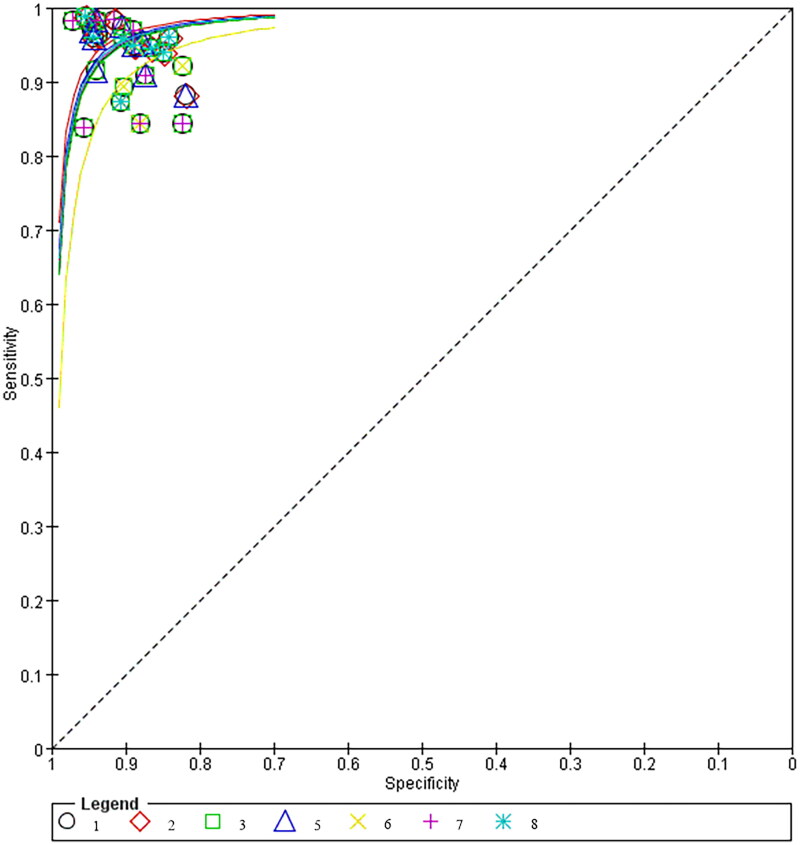
Summary combined SROC curves for different ultrasound signs.

#### 3.2.3. Funnel plot

**Figure 13.**
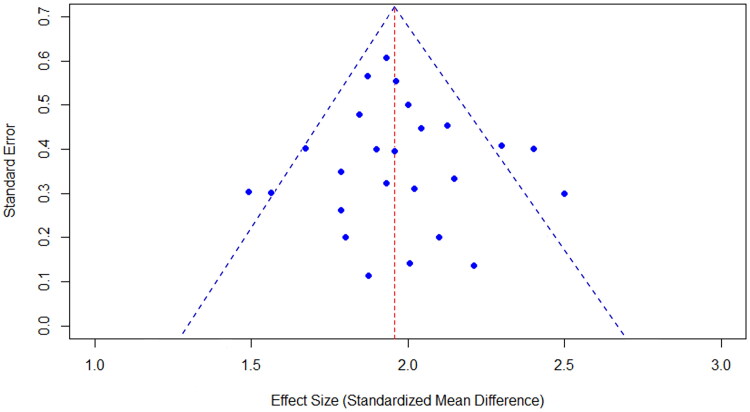
Funnel plot. Funnel plots of 25 included literature.

The funnel diagram of the 25 literatures included was shown in Figure 13. Literatures within 95% confidence interval account for the majority. Studies with large sample size and high precision were distributed at the top of the funnel plot and concentrated towards the middle. The funnel plot can directly reflect whether the estimated effect size of the original study was related to its sample size. There was publication bias, the funnel plot was asymmetrical and the distribution was skewed.

#### 3.2.4. Heterogeneity was analyzed by one-by-one exclusion method

**Figure 14.**
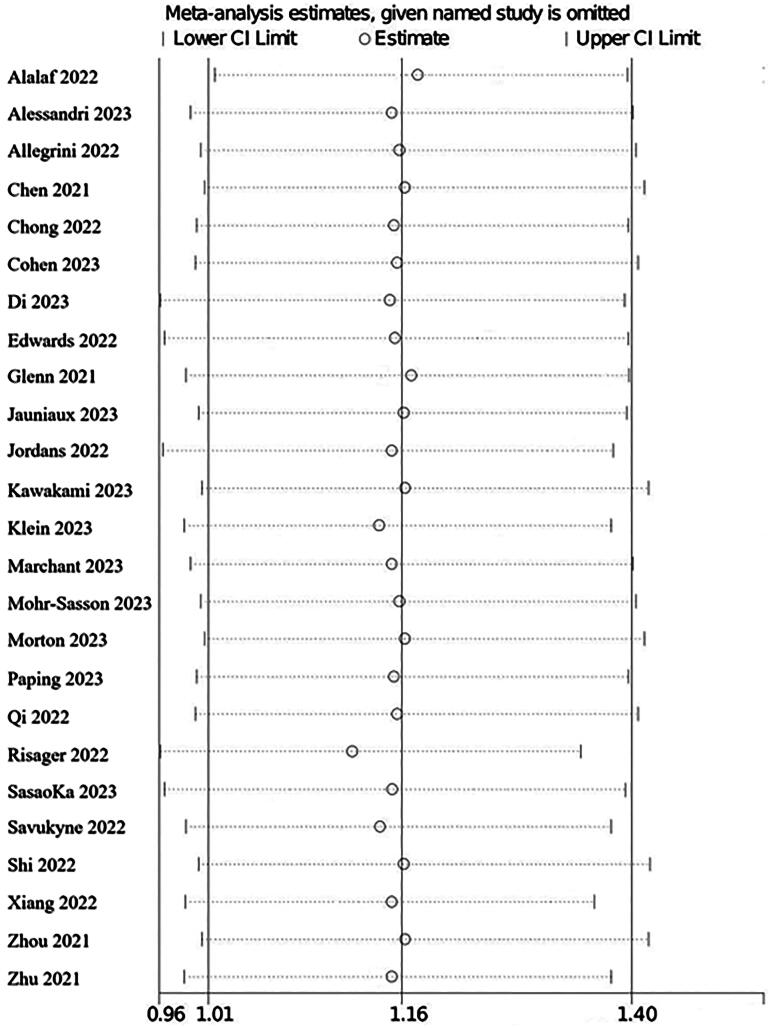
Heterogeneity analyzed by one-by-one exclusion method.

The 25 included literatures in this study were analyzed by one-by-one exclusion method, and the results showed that the heterogeneity analysis results of the remaining literatures were not large when any one of them was excluded, and the 95% confidence intervals of all the analyses were basically within the acceptable range, and were statistically significant (*p* < 0.05).shown in Figure 14.

## 4. Discussion

Cesarean section is recognised as a safe and effective mode of delivery in the face of high-risk pregnancies and late pregnancies that are not suitable for spontaneous delivery, and it significantly reduces maternal and fetal morbidity and mortality [44]. It has been reported that the cesarean section rate in developed countries is controlled at less than 40%, with an average cesarean section rate of about 20%, in contrast, the cesarean section rate across China is close to 50%, and the average cesarean section rate is much higher than the world average [45]. Under the impetus of China’s fertility policy, the cases of re-pregnancy with scarred uterus have increased, and the number of mothers who need a second caesarean section has risen, and under the influence of multiple factors, the trend of post-caesarean section complications has risen, among which poor healing of uterine scar and its complications should not be ignored [46]. There is a lack of clear consensus on the definition of post-caesarean section uterine scar healing dysplasia. In the relevant literature, it is also known as keloidal diverticulum, niche, keloidal defect, defect, incisional diverticulum, anechoic defect, etc. Scholars in the relevant fields prefer to use the term ‘keloidal diverticulum’ [47–49]. The incidence of keloid diverticula varies greatly between domestic and international studies, ranging from 7% to 70% [50]. During the formation of cesarean section scar, it usually goes through two stages: proliferative stage and mature stage. In the proliferative stage, the scar shows linear, red proliferative tissue with congested surface and filled with blood vessels inside, and the patient suffers from pain and itchiness, which usually lasts for 3 to 6 months [51]. Subsequently, it enters the mature stage, the degree of scar proliferation gradually reduces, the colour gradually approaches normal skin, and the pain and itching sensation gradually disappears [52]. However, cesarean section scar is not only a superficial skin trace, but also a reflection of deep tissue healing in the uterine area, cesarean section surgery causes trauma to the uterus, and cesarean section scar uterus is a normal physiological phenomenon, but for some women, it may be accompanied by pathological symptoms, such as uterine keloidal pregnancy, uterine keloidal diverticulum, placenta previa, uterine rupture, etc. [53], and these kinds of complications will affect women’s re fertility and physical health [54]. Therefore, the diagnosis of scar healing after cesarean section is crucial to determine the risk of potential complications in advance by observing and evaluating the scar.

Ultrasonography for scar healing after caesarean section is a non-invasive examination method that uses the propagation and reflection properties of ultrasound waves in human tissues to observe and assess the healing of uterine scar after caesarean section [55]. However, the relatively low resolution of conventional plain ultrasound images makes it difficult to clearly show the fine structure inside the scar, and the assessment of blood flow around the scar is not accurate enough to determine whether there are problems such as congestion or ischaemia in the scar [56]. In addition, ordinary ultrasound is easily interfered by factors such as intestinal gas and obesity, which affects the accuracy of the examination results. To overcome the limitations of ordinary ultrasound, the application of multimodal vaginal ultrasound in the detection of scar healing after cesarean section has gradually gained attention. Multimodal vaginal ultrasound combines a variety of technologies such as two-dimensional ultrasound, three-dimensional ultrasound, and colour Doppler, which is able to provide more comprehensive and accurate information about scar healing, not only displaying the morphology and structure of the scar with high definition, but also assessing the blood flow around the scar in real time, thus determine the healing status of the scar more accurately [57].

As a result of Meta-analysis in this study, 25 papers mentioned hypoechoic or anechoic pregnancy scar and absence of blood flow signal around the scar as a diagnostic ultrasound manifestation of poor healing after cesarean section, with a sensitivity of 92% and a specificity of 91%. Several studies have shown that hypoechoic or echo-less areas are highly correlated with poor scar healing after cesarean section. In ultrasound images, the normal myometrium usually exhibits homogeneous moderate echogenicity, and scar tissue is mainly composed of fibrous connective tissue, which lacks the structure of the normal myometrium internally, resulting in obstacles to the propagation of sound waves and the formation of a hypoechoic area. when the scar is poorly healed, the ultrasound probe will show that the scarred area hypoechoic or anechoic areas [58]. In the case of poor scar healing, there is usually a lack of significant blood flow signal around the scar area due to reduced vascular distribution or vascular occlusion, and several colour Doppler ultrasound studies have shown that the blood flow signal in poorly healed areas of the scar is significantly lower than that in normal tissue [59].

After caesarean section, ideal healing should show complete regeneration of the muscle layer at the incision site, restoring its original anatomical thickness and structural continuity. However, in cases of poor healing, ultrasound images may reveal significant thinning of the muscle layer at the incision site or even pathological changes such as fractures, discontinuities or defects. According to the results of the META analysis in this paper, a total of 11 publications indicated that these ultrasound features are important diagnostic criteria for poor scar healing, with a sensitivity of up to 95% and a specificity of 90%. Clinically, ultrasound observations revealed that the thickness of the muscle layer at the incision of poorly healed scar is usually lower than the normal physiological range, and the breaks or defects are clearly recognisable on ultrasound images [60].

Under normal conditions, uterine incisions appear as well-defined structures with clear edges and contours on ultrasound. When the scar heals poorly, the outline of the incision may become indistinct due to pathophysiological factors such as tissue proliferation, fibrosis or inflammation [61]. According to 22 publications in the META analysis, edge indistinctness is one of the key criteria for the diagnosis of poor scar healing, with a sensitivity of 99% and a specificity of 91%. By comparing the ultrasound images of a normal uterus and a poorly healed scarred uterus, the blurred contours and lack of definition of the latter incision were clearly observed.

Of the 25 papers included, 9 indicated irregularity in lesion morphology as a diagnostic criterion for poor scar healing, and combined analysis showed a sensitivity of 94% and a specificity of 91%. In ultrasound images, the lesion morphology in areas of poor scar healing often appears as irregular areas of hypoechoic or mixed echoes, and this irregularity may be due to non-homogeneity and structural disorganisation of the scar tissue during the healing process [62].

In addition, echogenic enhancement and thickness inhomogeneity of the myometrium in the lower uterine segment are ultrasound features of poor scar healing. Six of the 25 papers included used this as a diagnostic criterion, with a sensitivity of 90 per cent and a specificity of 88 per cent in the combined analysis. Ultrasound observations show that the myometrial echoes in the lower segment of the uterus in poorly healed scar usually show heterogeneous enhancement, and myometrial thickness shows significant variability in different regions [63].

The thickness of the lower segment of the uterus is a key parameter in assessing uterine recovery after caesarean section. Normally, the thickness of the lower uterine segment should be greater than 3.73 mm.According to nine publications in META analysis, a thickness of ≤3.73 mm of the lower uterine segment is a diagnostic criterion for poor scar healing, with a sensitivity of 90% and a specificity of 92% in the combined analysis. Meanwhile, myometrial endometrial thickness ≤1.5 mm was also considered as a diagnostic criterion for poor scar healing by 8 papers, with a sensitivity of 95% and a specificity of 91% in the combined analysis.

Multimodal vaginal ultrasound has high accuracy in evaluating cesarean section scar healing, but it is dependent on skilled operators, human factors have a great impact on the heterogeneity of results, different operators have different judgments of ultrasound images, and it is difficult to unify the criteria of operators in clinical practice, which leads to great heterogeneity of ultrasound results. The implementation of MTVUS in routine clinical practice requires professional training of the operator, as far as possible to unify the operator’s operation and image reading, and should calculate the cost and clarify the work flow. The work process is unified and the details are strictly controlled. In this study, the dots in the funnel plot are scattered, and some dots are written outside the confidence interval, and the whole plot presents a skewed distribution. This suggests that publication bias exists in our meta-analysis and should be taken seriously. In future studies, Egger test and Begg test will be used to find out the specific data of the asymmetry of the funnel plot to help readers fully understand this research. Patient-centered research is the core of future uterine scar research, and studies on patient comfort with MTVUS, C-section acceptance, and uterine scar care require researchers to strengthen communication with patients and closely follow up the perioperative care of patients from before delivery. Suture materials used in cesarean section incision have a great impact on wound healing. We will subdivide and analyze suture materials in future studies. By deepening these studies, we will be able to identify the long-term outcomes of uterine scar healing and find standardized protocols for uterine scar healing. This is helpful to the development of clinical caesarean section surgery and suture technology, and has a clear guiding effect on postpartum scar care. This could be a boon to all mothers.

## 5. Conclusion

In this paper, the application of multimodal vaginal ultrasound in the detection of scar healing after caesarean section and its accuracy were investigated by comprehensively analysing 25 papers. The results showed that multimodal vaginal ultrasound, as a non-invasive, high-resolution examination method, can effectively assess the healing status of uterine scar after caesarean section, and especially shows high sensitivity and specificity in identifying the ultrasound features of poor scar healing. By comprehensively analysing ultrasound features such as hypoechoic or echo-less areas, absence of blood flow signals around the scar, thinning of the myometrium at the incision, poorly defined margins, irregular morphology of the lesion, and enhanced echogenicity and thickness heterogeneity of the myometrium of the lower uterine segments, the present study provides clinicians with a reliable tool to more accurately diagnose and monitor poorly healing post-caesarean section keloidal scars and their potential complications. In addition, the study highlights the importance of the thickness of the lower uterine segment and the thickness of the endometrium of the myometrium as important parameters in the assessment of scar healing. Multimodal vaginal ultrasound technology has shown significant clinical value in the detection and assessment of scar healing after caesarean section, helping to optimise patient management strategies, reduce the risk of complications and provide safer guidance for women planning another pregnancy. Although this study provides valuable information for the ultrasound assessment of scar healing after cesarean delivery, there are some limitations, such as the interpretation of ultrasound images with multimodal vaginal ultrasound technology still depends on the experience and skills of the operator, with a high degree of study variability, and the META analysis provides a way to quantify the results of different studies, but the selection of statistical models, the merging of data and the analyses may be limitations that may affect the results. The difference of operators and the heterogeneity of studies have a certain impact on the diagnosis of uterine scar, and may also affect the clinical prevention and treatment guidelines. Therefore future studies should be explored more extensively and in depth to further improve the value of multimodal vaginal ultrasound in clinical practice.

## Supplementary Material

PRISMA_2020_checklist.docx

## Data Availability

The datasets used and/or analyzed during the current study are available from the corresponding author on reasonable request.
